# Toward a universal treatment for cancer: cell inflation assisted chemotherapy

**DOI:** 10.1002/cam4.91

**Published:** 2013-05-28

**Authors:** Daniel Corcos

**Affiliations:** INSERM U955, Faculté de Médecine de Créteil8 Rue du Général Sarrail, 94010, Créteil Cedex, France

**Keywords:** Cancer chemotherapy, granulocytes, myelosuppression, negative feedback

## Abstract

Cancers show considerable genetic and functional heterogeneity, preventing the development of a universal anticancer drug. Here, I argue that it is nevertheless possible to elaborate a therapeutic strategy that can be used in almost every cancer, exploiting the negative feedback effect of normal cells on the proliferation of their precursors. This method, termed cell inflation assisted chemotherapy, is aimed at blocking normal cell division prior to high-dose antimitotic chemotherapy. Evidence for a negative feedback effect on granulocyte production suggests that it is possible to prevent neutropenia by transfusion of autologous granulocytes. In a first step, this protocol will be devised to protect neutrophils and to prevent granulopenia in patients treated with intensive chemotherapy. In its simplest form, it will consist of a leukapheresis–storage–reinjection sequence just prior to drug administration. Then, if the proof of concept is established, a more systematic use of intensive cell cycle-specific chemotherapy, together with protection of other lineages through temporary mitotic blockade might be a treatment applicable for most cancers.

Negative feedback effect of normal cells on the proliferation of their precursors may be used to protect them from high-dose antimitotic chemotherapy, preventing myelosuppression. In its simplest form, cell inflation assisted chemotherapy will consist of a leukapheresis-storage-reinjection sequence just prior to drug administration.

## Introduction

Cancer is a collection of different diseases with a common principle: uncontrolled cell growth. Heterogeneity between and within these diseases seems to preclude any universal treatment against cancer. However, based on the unifying principle of cancer, uncontrolled growth, it is possible to devise a strategy which could be used for every cancer.

Conventional anticancer drugs work by killing dividing cells. The effect of these drugs is not specific for cancer cells, but is also observed for highly dividing normal cells. An obvious idea is that, if normal cells could be reversibly prevented from dividing during the time of chemotherapy, they would be protected 1. Therefore, temporarily blocking normal cell division could make chemotherapy considerably safer. However, the cytopenia that would be the consequence of mitotic blockade would not be desirable.

**Figure 1:**
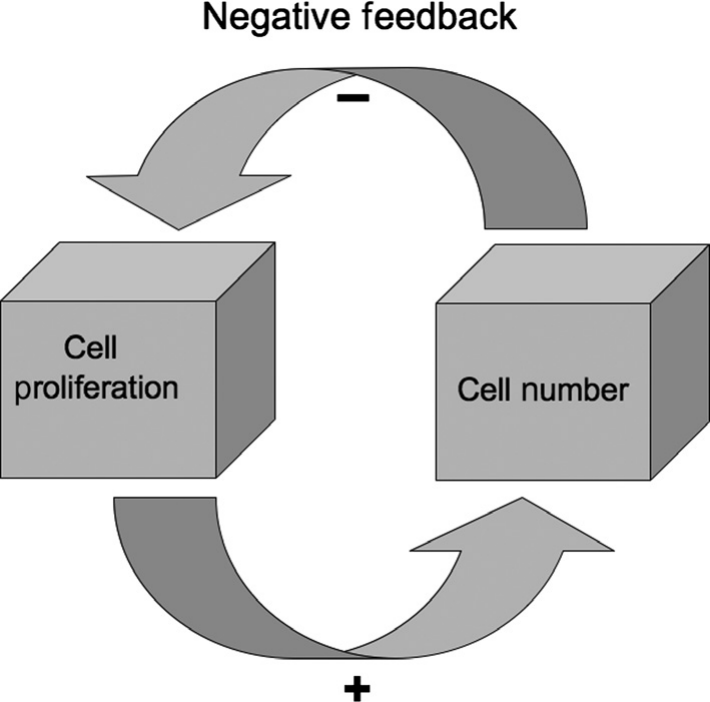
A very simple representation of negative feedback in the control of cell number. Note that the forward arrow indicates an obligatory consequence of cell proliferation, whereas the backward arrow is specific for regulation. Many vertebrate cell populations contain actually a stem cell compartment, a proliferative pool, and terminally differentiated cells, which have important kinetic implications, but does not change the overall model. An important feature of negative feedback is cell-type specificity.

For many tissues, cells are produced in order to compensate cell loss. This may mean that in the presence of an excess of cells, cell division would not occur (negative feedback; Fig. 1). Thus, it can be predicted that, if it is possible to obtain «cell inflation» before chemotherapy, normal progenitor cells would be protected in a tissue-specific manner.

Before going further, one has to consider that all anticancer chemotherapy side effects are not related to the killing of dividing cells. In fact, as the rate of cell division within a tumor is generally less important than that in bone marrow and in the gastrointestinal tract 2, other drugs have been selected empirically for their relative specificity toward cancer cells. Trends in anticancer therapy have therefore resulted in the usage of drugs with weaker effects on normal cell division. However, if it were proven that normal dividing cells can be easily protected, then, interest in using more widely drugs that are cell cycle-specific would be regained.

## In many tissues, cell production rates are negatively regulated by the numbers of differentiated cells

It has been known for a long time that some tissues can regenerate after cell loss. Regeneration stops when a certain number of cells, close to the physiological number, is reached 3. Both processes, regeneration and its termination, are indicative of a negative feedback loop. For tissues that are renewed daily from progenitors, similarly, negative feedback is the likely mechanism for adapting cell production to cell loss, and in some cases, this has been demonstrated.

Negative feedback can be related to the inhibition of a growth-promoting pathway as a function of the number of cells. Soluble growth inhibitors that act on the specific tissue that secretes them have been termed chalones. Although, for a long time, chalones have been sought without success, in recent years, growth inhibitors with the property of chalones have been identified. Bone morphogenetic protein 4 and myostatin 5 have been identified as chalones for hair follicles and muscle, respectively. Neutrophil elastase has been proposed to act as a neutrophil chalone by antagonizing the effect of granulocyte–colony-stimulating factor (G-CSF) 6–7.

Inhibition of a growth-promoting pathway is more often achieved by decreasing growth factor production or availability. Inhibition of erythropoietin production by oxygen is a way of ensuring feedback for red cell production 8. One way frequently used is the clearance of a growth factor by the cells. This mechanism is involved in thrombopoiesis 9. It has been shown that thrombopoietin (Tpo) levels are controlled by platelet numbers through Mpl-mediated Tpo uptake and degradation. Similarly, it has been proposed that IL-7 accumulates in CD4 lymphopenia as a consequence of diminished use 10.

Whatever the mechanism involved, negative feedback could be theoretically used to block mitosis as a consequence of «cell inflation», in order to protect normal cells from the effect of antimitotic drugs. However, obtaining an excess of normal cells for each lineage would be difficult to achieve, and in addition, may not be necessary, as the most important and limiting effect of antimitotic chemotherapy is myelosuppression, and more specifically neutropenia. Therefore, if a temporary suspension of granulocyte progenitor division is possible, then it can be expected that this life-threatening side effect of chemotherapy would be prevented.

## Evidence for negative feedback in the control of granulocyte production

It is thus important to evaluate the evidence that granulocyte production is regulated by negative feedback and the prospects that granulocyte excess (cell inflation) would reduce progenitor division rate. Arguments for negative feedback have been provided by studies in dogs and mice: neutropenia induced by leukapheresis, antiserum, or irradiation 11 appears to increase the rate of production of granulocyte precursors. Cyclic neutropenia, a disease characterized by oscillation of blood neutrophil counts with periods of severe neutropenia occurring every 21 days, is also considered as providing evidence for negative feedback 12. Most mathematical models of granulopoiesis include a feedback mechanism 13. Currently, the best documented mechanism for explaining negative feedback in granulopoiesis is increased clearance of G-CSF by granulocytes, which may involve receptor-mediated removal 14,15. G-CSF levels increase following chemotherapy-induced neutropenia, consistent with this model, but they tend to decrease before blood neutrophil counts rise again 17, indicating a more complex relationship between G-CSF and blood granulocyte numbers.

## Usefulness of protecting granulocytes during chemotherapy

Although protecting normal cells from chemotherapy seems to be a reasonable goal (see for instance 18), translating this goal into changes in therapeutic options may not be straightforward. First, in current clinical practice, neutropenia is only one of the side effects of chemotherapies, and its consequences can be managed to some extent with antibiotic prophylaxy. This criticism can be overcome by the consideration that myelosuppression has set the limits of chemotherapy doses. Second, a major obstacle for intensifying chemotherapy in cancer treatment is the poor perception of high-dose chemotherapy as a treatment of metastasized cancer by the medical community. In therapeutic trials, myelosuppression is circumvented by the use of autologous hematopoietic stem cell transplantation (HSCT). This interferes with the chemotherapy protocol, as autologous HSCT cannot be repeated several times 19, whereas in conventional chemotherapy, repetition of cycles is important in recruiting new cycling tumor cells, which become the targets in the next chemotherapy round. It is therefore likely that the gain in increasing the dose of chemotherapy is counterbalanced by the decrease in efficiency due to the low number of cycles. In addition, transplantation of HSCT is at risk of contamination with cancer cells 20. This is of concern because chemotherapy is not used after transplantation. Another issue is the use of hematopoietic growth factors in these protocols 21, which might have unexpected adverse effects on tumor growth. Finally, the temporary neutropenia induced by high-dose chemotherapy may have adverse effects, not limited to infections. In fact, there is increasing evidence that neutrophils have a potent antitumoral activity 22,23. Thus, the poor antitumor efficiency associated with intensive chemotherapy, leading to the abandonment of this treatment in many situations, might be circumvented by protection of the neutrophil lineage. Therefore, although the supporters of current protocols for high-dose chemotherapy 25 are the minority, it does not mean that intensifying chemotherapy would not be efficient in different settings.

## Technical issues for blocking granulopoiesis prior to chemotherapy

Here, we will discuss the prospects for obtaining cell inflation in granulocytes. Two main possibilities exist: homologous and autologous transfusion. Homologous transfusion would have the advantage of providing large quantities of cells. However, this method would preferably require human leukocyte antigen (HLA) compatibility, limiting its use. In addition, immunological responses, even in HLA-matched individuals, may be a source of side effects. Autologous transfusion would be considerably safer. Then, two options could be considered: transfer of in vitro differentiated granulocytes obtained from hematopoietic precursor cells 26 or transfusion of stored leukapheresis-derived cells after normalization of the granulocyte pool. Transfer of large numbers of in vitro derived granulocytes is not yet feasible. In addition to the technical difficulties, this method would also have the inconvenience of delaying chemotherapy. In contrast, transfusion of leukapheresis-derived granulocytes after storage would not delay treatment much and might not require further sophisticated optimization. The only prerequisite for autotransfusion would be the time of restoration of the cell pool being shorter than the storage time.

For erythrocytes, this requirement has been fulfilled 27. In the protocol of Damsgaard et al. 27, one fifth of the blood is withdrawn and packed red cells are reinjected on the 28th day. At that time, before reinfusion, the hematocrit level is back to normal. After reinfusion, the hematocrit increases, which corresponds to a situation of red cell inflation. Reticulocyte counts are reduced by 25–37% from day 7 to 21 after reinfusion, indicating a strong effect of red cell inflation on erythrocyte production. However, the aim of this experiment was to detect autologous blood doping procedures, and so no information was given regarding the decrease in mitosis in the bone marrow. If we take the hypothesis that the negative feedback effect is almost immediate, then there should be a period of time when there is almost complete suppression of mitosis.

Upon leukapheresis, large numbers of granulocytes can be collected 28–29. In granulocyte donation protocols, dexamethasone and/or G-CSF are used. After dexamethasone stimulation, the number of white blood cells that are collected in 3 h is around 30 × 10^9^. However, in the context of cell inflation assisted chemotherapy, it might be safer to perform leukapheresis without stimulation. In exceptional situations, when urgent granulocyte transfusion is performed for neonates, 10 × 10^9^ white blood cells can be collected (D. Lemau de Talancé, pers. comm.; Atallah and Schiffer 29), the majority of them being granulocytes. Taking the size of the total granulocyte pool as being 38 × 10^9^ cells 30, this means that nearly a fifth of this pool can be stored and used for feedback, which is similar to the situation fulfilled for erythrocytes in Damsgaard et al.'s protocol.

Granulocytes have a much shorter storage time (around 10 h) than erythrocytes, but granulocyte production can rapidly increase upon stimulation 31–32. Granulocyte half-life is much shorter than that of erythrocytes 30–33, indicating that the dynamics of cell production is much faster.

Therefore, the critical point in a leukapheresis–storage–reinjection protocol might be the restoration of the granulocyte pool during the 10 h storage time. This has to be tested experimentally in healthy volunteers. Another point to be determined experimentally is the efficiency and the time course of mitosis suppression after reinjection. Since such determination would require bone marrow examination, animal experimentation (preferably on primates) would be necessary.

In the case that mitosis suppression were unsatisfactory, related to insufficient granulocyte restoration due to the short time before reinjection, it would be necessary to find a means to extend granulocyte storage time. One possible way to increase granulocyte half-life *ex vivo* would be to add glucocorticoids 34–35 in the collection bag. The effect of G-CSF might be tested, but then it would be necessary to check that all the G-CSF has been consumed before reinjecting, and that this does not lead to an important decrease in G-CSF receptor levels at the granulocyte surface, given that receptor-mediated removal is involved in G-CSF clearance.

Another possibility is to freeze granulocytes. Although frozen granulocytes are not used in granulocyte donation, freezing of granulocytes allows the recovery of a large number of viable cells after thawing 36. This method has yet to be optimized, but would have the advantage of allowing full granulocyte pool restoration before reinjection. Another promising future direction would be the use of granulocytes generated from hematopoietic progenitor cells 26.

Another point to discuss is the safety of the procedure: data from the literature indicate that an excess of granulocytes would be well tolerated 37. An issue of concern might be the presence of dead cells and cytokines in the collection bag, but as granulocyte donation is performed routinely with large numbers of granulocytes without severe side effect, it is likely that the leukapheresis–storage–reinjection protocol will be safe.

From the above considerations, it appears that it may be feasible to reduce the mitotic rate of granulocyte progenitors safely and possibly without sophisticated technology. Some difficulties, like extending granulocyte storage time, may require some effort, but would not be very challenging. Therefore, a protocol using granulocyte inflation immediately before chemotherapy may be protective against neutropenia and could be useful in a limited number of cancers, where chemotherapy frequently leads to myelosuppression.

## A cure for all cancers?

How can we get from prevention of myelosuppression to a universal treatment for cancer? First, it is important to remember that the first hint suggesting that some compounds may have anticancer activity came from the fact that they caused leukopenia 38. The relationship between broad antimitotic activity and leukopenia is strong, given that granulocytes are rapidly renewed. But as the mitotic rate of white cells is often higher than that of tumors, the efficiency of chemotherapy usually relies on a second source of specificity. This specificity is paid in the form of side effects unrelated to antimitotic effects. Even with this reduction in the risk of myelosuppression, febrile neutropenia after systemic chemotherapy is still a cause of mortality in many types of cancer 39. Evidence of the usefulness of cellular inflation assisted chemotherapy in specific cancers, for which myelosuppressive drugs are used, should lead to a change in the type of chemotherapy that is used in other cancers, resulting in the use of drugs that more specifically kill mitotic cells. Granulocyte autotransfusion, as considered above, therefore has a great potential in increasing survival after chemotherapy in many cancers.

With protection of the granulocyte lineage by inflation, other side effects of intensive chemotherapy may appear. Myelosuppression-related thrombopenia could be prevented by platelet inflation before chemotherapy, through storage and reinjection of autologous platelet concentrates. Anemia may be prevented by compatible homologous red cell transfusion prior to chemotherapy.

Currently, it is difficult to think of a cell inflation protocol that could protect the cells of the gastrointestinal tract from antimitotic drugs, but finding a way to temporarily «freeze» their cell cycle may be an aim of future research. Protecting all normal dividing cells would be an almost impossible task, but all that is needed would be to protect the cell types whose loss causes chemotherapy side effects. In the future, this might be easier than targeting any oncogenic mutation or signaling pathway in heterogeneous cancer populations, as would be required for genotype-directed cancer therapy 40.

## Conclusion and Future Perspectives

By protecting normal dividing cells using cell inflation-induced negative feedback, it may be possible to cure a large number of cancers after setting up the method through a step-by-step approach (Table 1). Although present day intensive chemotherapy has poor antitumor efficiency in many situations, it is likely that many therapeutic failures are related to the inability to perform several cycles, due to hematologic side effects, or to the inability to repeat HSCT. Protecting granulocytes by a cell inflation protocol would be a significant progress in the use of high-dose chemotherapy.

**Table:** Steps for setting up cell inflation assisted chemotherapy

(1) **Proof of concept** of operational negative feedback of granulocytes on the division of progenitors and determination of the kinetics of mitosis inhibition (primates).
(2) **Feasibility study of granulocyte inflation in human**. Determination of the optimal time for restoration of the granulocyte pool before reinjection. Storage time of human granulocytes, with preservation of efficient negative feedback. Effect of freezing on negative feedback (primates and healthy volunteers).
(3) **Search for evidence of efficacy** of prior granulocyte inflation in preventing myelosuppression induced by chemotherapy in cancers at high risk of febrile neutropenia (selected cancer patients).
(4) **Switch to myelosuppressive drugs** in cancers where other drugs are usually preferred (numerous cancer patients).
(5) **Setting up cell inflation protocols** for other cell types.

For future prospects, the ability to discriminate cancer cells from normal cells solely on the basis of proliferation offers a way to deal with tumor heterogeneity. Acquired resistance to traditional as well as targeted chemotherapy is dependent on selection of cancer cell subpopulations. Combination therapies are the best way to prevent the emergence of resistant cell clones 41. With personalized targeted chemotherapies, a problem will appear related to the side effects of multiple drug interaction and the difficulty of performing randomized trials, given the uniqueness of each tumor. Another important issue is the lack of knowledge on crosstalk circuits between signaling pathways 40. Traditional chemotherapy has the advantage over targeted therapy that side effects are more predictable, and it would be possible in theory to select agents with side effects restricted to dividing cells. Currently, traditional chemotherapy uses cell cycle-nonspecific as well as cell cycle-specific drugs. Although cell cycle-specific drugs seem to be better suited for normal cell protection by cell inflation, the method could be used with cell cycle-nonspecific drugs, as they also preferentially kill highly dividing cells. However, the ultimate goal would be to devise combinational therapy with cell cycle-specific drugs where the only way for cancer cells to escape death would be to have slower growth.

It is important to stress that this strategy is not incompatible with the use of, and could benefit from, additional targeted chemotherapy.

Finally, an international effort is needed to carry out the adjustments and development required for optimizing negative feedback (i.e., cell sorting and storage) and adapting intensive antimitotic chemotherapy.

## References

[b1] Blagosklonny MV, Pardee AB (2001). Exploiting cancer cell cycling for selective protection of normal cells. Cancer Res.

[b2] Reiskin AB, Mendelsohn ML (1964). A comparison of the cell cycle in induced carcinomas and their normal counterpart. Cancer Res.

[b3] Lui JC, Baron J (2011). Mechanisms limiting body growth in mammals. Endocr. Rev.

[b4] Plikus MV, Mayer JA, Baker D, de la Cruz RE, Maini PK, Maxson R (2008). Cyclic dermal BMP signalling regulates stem cell activation during hair regeneration. Nature.

[b5] Lee SJ (2004). Regulation of muscle mass by myostatin. Annu. Rev. Cell Dev. Biol.

[b6] Horwitz M, Benson KF, Duan Z, Person RE, Wechsler J, Williams K (2003). Role of neutrophil elastase in bone marrow failure syndromes: molecular genetic revival of the chalone hypothesis. Curr. Opin. Hematol.

[b7] El Ouriaghli F, Fujiwara H, Melenhorst JJ, Sconocchia G, Hensel N, Barrett AJ (2003). Neutrophil elastase enzymatically antagonizes the in vitro action of G-CSF: implications for the regulation of granulopoiesis. Blood.

[b8] Eckardt KU, Kurtz A (2005). Regulation of erythropoietin production. Eur. J. Clin. Invest.

[b9] Tiedt R, Coers J, Ziegler S, Wiestner A, Hao-Shen H, Bornmann C (2009). Pronounced thrombocytosis in transgenic mice expressing reduced levels of Mpl in platelets and terminally differentiated megakaryocytes. Blood.

[b10] Fry TJ, Mackall CL (2005). The many faces of IL-7: from lymphopoiesis to peripheral T cell maintenance. J. Immunol.

[b11] Morley A, Stohlman F (1970). Studies on the regulation of granulopoiesis. I. The response to neutropenia. Blood.

[b12] von Schulthess GK, Mazer NA (1982). Cyclic neutropenia (CN): a clue to the control of granulopoiesis. Blood.

[b13] Ostby I, Winther R (2004). Stability of a model of human granulopoiesis using continuous maturation. J. Math. Biol.

[b14] Layton JE, Hockman H, Sheridan WP, Morstyn G (1989). Evidence for a novel in vivo control mechanism of granulopoiesis: mature cell-related control of a regulatory growth factor. Blood.

[b15] Roskos LK, Lum P, Lockbaum P, Schwab G, Yang BB (2006). Pharmacokinetic/pharmacodynamic modeling of pegfilgrastim in healthy subjects. J. Clin. Pharmacol.

[b16] Foley C, Mackey MC (2009). Mathematical model for G-CSF administration after chemotherapy. J. Theor. Biol.

[b17] Watari K, Asano S, Shirafuji N, Kodo H, Ozawa K, Takaku F (1989). Serum granulocyte colony-stimulating factor levels in healthy volunteers and patients with various disorders as estimated by enzyme immunoassay. Blood.

[b18] Adair JE, Beard BC, Trobridge GD, Neff T, Rockhill JK, Silbergeld DL (2012). Extended survival of glioblastoma patients after chemoprotective HSC gene therapy. Sci. Transl. Med.

[b19] Stadtmauer EA, O'Neill A, Goldstein LJ, Crilley PA, Mangan KF, Ingle JN (2000). Conventional-dose chemotherapy compared with high-dose chemotherapy plus autologous hematopoietic stem-cell transplantation for metastatic breast cancer. Philadelphia Bone Marrow Transplant Group. N. Engl. J. Med.

[b20] Preti RA, Lazarus HM, Winter J, Stadtmauer EA, Nadasi S, McMannis J (2001). Tumor cell depletion of peripheral blood progenitor cells using positive and positive/negative selection in metastatic breast cancer. Cytotherapy.

[b21] Bakanay SM, Demirer T (2012). Novel agents and approaches for stem cell mobilization in normal donors and patients. Bone Marrow Transplant.

[b22] Souto JC, Vila L, Bru A (2011). Polymorphonuclear neutrophils and cancer: intense and sustained neutrophilia as a treatment against solid tumors. Med. Res. Rev.

[b23] Granot Z, Henke E, Comen EA, King TA, Norton L, Benezra R (2011). Tumor entrained neutrophils inhibit seeding in the premetastatic lung. Cancer Cell.

[b24] Hicks AM, Riedlinger G, Willingham MC, Alexander-Miller MA, Pettenati C, von Kap-Herr MJ (2006). Transferable anticancer innate immunity in spontaneous regression/complete resistance mice. Proc. Natl. Acad. Sci. USA.

[b25] Martino M, Bottini A, Rosti G, Generali D, Secondino S, Barni S (2012). Critical issues on high-dose chemotherapy with autologous hematopoietic progenitor cell transplantation in breast cancer patients. Expert Opin. Biol. Ther.

[b26] Timmins NE, Nielsen LK (2009). Blood cell manufacture: current methods and future challenges. Trends Biotechnol.

[b27] Damsgaard R, Munch T, Morkeberg J, Mortensen SP, Gonzalez-Alonso J (2006). Effects of blood withdrawal and reinfusion on biomarkers of erythropoiesis in humans: implications for anti-doping strategies. Haematologica.

[b28] Price TH (2007). Granulocyte transfusion: current status. Semin. Hematol.

[b29] Atallah E, Schiffer CA (2006). Granulocyte transfusion. Curr. Opin. Hematol.

[b30] Galbraith PR, Valberg LS, Brown M (1965). Patterns of granulocyte kinetics in health, infection and in carcinoma. Blood.

[b31] Lieschke GJ, Grail D, Hodgson G, Metcalf D, Stanley E, Cheers C (1994). Mice lacking granulocyte colony-stimulating factor have chronic neutropenia, granulocyte and macrophage progenitor cell deficiency, and impaired neutrophil mobilization. Blood.

[b32] Dale DC (1998). The discovery, development and clinical applications of granulocyte colony-stimulating factor. Trans. Am. Clin. Climatol. Assoc.

[b33] Pillay J, Vrisekoop I, den Braber N, Kwast LM, Borghans RJ, de Boer JA (2010). In vivo labeling with 2H_2_O reveals a human neutrophil lifespan of 5.4 days. Blood.

[b34] Cox G (1995). Glucocorticoid treatment inhibits apoptosis in human neutrophils. Separation of survival and activation outcomes. J. Immunol.

[b35] Liles WC, Dale DC, Klebanoff SJ (1995). Glucocorticoids inhibit apoptosis of human neutrophils. Blood.

[b36] Akkok CA, Liseth K, Hervig T, Ryningen A, Bruserud O, Ersvaer E (2009). Use of different DMSO concentrations for cryopreservation of autologous peripheral blood stem cell grafts does not have any major impact on levels of leukocyte- and platelet-derived soluble mediators. Cytotherapy.

[b37] D'Souza A, Jaiyesimi I, Trainor L, Venuturumili P (2008). Granulocyte colony-stimulating factor administration: adverse events. Transfus. Med. Rev.

[b38] Hirsch J (2006). An anniversary for cancer chemotherapy. JAMA.

[b39] Kuderer NM, Dale DC, Crawford J, Cosler LE, Lyman GH (2006). Mortality, morbidity, and cost associated with febrile neutropenia in adult cancer patients. Cancer.

[b40] Bernards R (2012). A missing link in genotype-directed cancer therapy. Cell.

[b41] Woodcock J, Griffin JP, Behrman RE (2011). Development of novel combination therapies. N. Engl. J. Med.

